# Developing an Electroencephalography-Based Model for Predicting Response to Antidepressant Medication

**DOI:** 10.1001/jamanetworkopen.2023.36094

**Published:** 2023-09-28

**Authors:** Benjamin Schwartzmann, Prabhjot Dhami, Rudolf Uher, Raymond W. Lam, Benicio N. Frey, Roumen Milev, Daniel J. Müller, Pierre Blier, Claudio N. Soares, Sagar V. Parikh, Gustavo Turecki, Jane A. Foster, Susan Rotzinger, Sidney H. Kennedy, Faranak Farzan

**Affiliations:** 1 eBrain Lab, School of Mechatronic Systems Engineering, Simon Fraser University, Surrey, British Columbia, Canada; 2 Department of Psychiatry, University of Toronto, Toronto, Ontario, Canada; 3 Centre for Addiction and Mental Health, Toronto, Ontario, Canada; 4 Department of Psyciatry, Dalhousie University, Halifax, Nova Scotia, Canada; 5 Department of Psychiatry, University of British Columbia, Vancouver, British Columbia, Canada; 6 Department of Psychiatry and Behavioural Neurosciences, McMaster University, Hamilton, Ontario, Canada; 7 Mood Disorders Program and Women’s Health Concerns Clinic, St. Joseph’s Healthcare, Hamilton, Ontario, Canada; 8 Department of Psychiatry, Queen’s University, Providence Care, Kingston, Ontario, Canada; 9 Department of Psychology, Queen’s University, Providence Care, Kingston, Ontario, Canada; 10 Mood Disorders Research Unit, University of Ottawa Institute of Mental Health Research, Ottawa, Ontario, Canada; 11 University of Michigan Depression Center, Ann Arbor; 12 Douglas Mental Health University Institute, Department of Psychiatry, McGill University, Montreal, Quebec, Canada; 13 Department of Psychiatry and Behavioural Neurosciences, McMaster University, Hamilton, Ontario, Canada; 14 Center for Depression Research and Clinical Care, University of Texas Southwestern Medical Center, Dallas; 15 Unity Health Toronto, Toronto, Ontario, Canada

## Abstract

**Importance:**

Untreated depression is a growing public health concern, with patients often facing a prolonged trial-and-error process in search of effective treatment. Developing a predictive model for treatment response in clinical practice remains challenging.

**Objective:**

To establish a model based on electroencephalography (EEG) to predict response to 2 distinct selective serotonin reuptake inhibitor (SSRI) medications.

**Design, Setting, and Participants:**

This prognostic study developed a predictive model using EEG data collected between 2011 and 2017 from 2 independent cohorts of participants with depression: 1 from the first Canadian Biomarker Integration Network in Depression (CAN-BIND) group and the other from the Establishing Moderators and Biosignatures of Antidepressant Response for Clinical Care (EMBARC) consortium. Eligible participants included those aged 18 to 65 years who had a diagnosis of major depressive disorder. Data were analyzed from January to December 2022.

**Exposures:**

In an open-label trial, CAN-BIND participants received an 8-week treatment regimen of escitalopram treatment (10-20 mg), and EMBARC participants were randomized in a double-blind trial to receive an 8-week sertraline (50-200 mg) treatment or placebo treatment.

**Main Outcomes and Measures:**

The model’s performance was estimated using balanced accuracy, specificity, and sensitivity metrics. The model used data from the CAN-BIND cohort for internal validation, and data from the treatment group of the EMBARC cohort for external validation. At week 8, response to treatment was defined as a 50% or greater reduction in the primary, clinician-rated scale of depression severity.

**Results:**

The CAN-BIND cohort included 125 participants (mean [SD] age, 36.4 [13.0] years; 78 [62.4%] women), and the EMBARC sertraline treatment group included 105 participants (mean [SD] age, 38.4 [13.8] years; 72 [68.6%] women). The model achieved a balanced accuracy of 64.2% (95% CI, 55.8%-72.6%), sensitivity of 66.1% (95% CI, 53.7%-78.5%), and specificity of 62.3% (95% CI, 50.1%-73.8%) during internal validation with CAN-BIND. During external validation with EMBARC, the model achieved a balanced accuracy of 63.7% (95% CI, 54.5%-72.8%), sensitivity of 58.8% (95% CI, 45.3%-72.3%), and specificity of 68.5% (95% CI, 56.1%-80.9%). Additionally, the balanced accuracy for the EMBARC placebo group (118 participants) was 48.7% (95% CI, 39.3%-58.0%), the sensitivity was 50.0% (95% CI, 35.2%-64.8%), and the specificity was 47.3% (95% CI, 35.9%-58.7%), suggesting the model’s specificity in predicting SSRIs treatment response.

**Conclusions and Relevance:**

In this prognostic study, an EEG-based model was developed and validated in 2 independent cohorts. The model showed promising accuracy in predicting treatment response to 2 distinct SSRIs, suggesting potential applications for personalized depression treatment.

## Key Points

**Question:**

Can a robust and generalizable electroencephalography-based model be developed to predict whether a patient with depression will benefit from specific medication treatments?

**Findings:**

In this prognostic study, a model based on electroencephalography predicted response to escitalopram for a cohort of 125 patients with an accuracy of 64.2%. It was validated in an independent sample of 105 patients, in which the model demonstrated an accuracy of 63.7% in predicting response to sertraline.

**Meaning:**

These findings suggest that the use of electroencephalography can provide a reliable method for predicting response to specific antidepressant medications and may help match patients with depression to optimized treatment.

## Teaser

This prognostic study explores the use of an electroencephalography (EEG)–based model to predict response to specific selective serotonin reuptake inhibitor treatments for major depressive disorder.

## Introduction

Major depressive disorder (MDD) is a leading cause of disability worldwide, affecting more than 300 million people.^1^ Antidepressant medications are prescribed as a first-line treatment, but remission rates after the first and second trials range from 35% to 50%^2,3^ and decline further with subsequential trials.^2^ Given that each trial may take up to 12 weeks, predicting medication response could greatly reduce illness duration and associated negative outcomes.

In the past 2 decades, many studies have assessed the utility of electroencephalography (EEG) for identifying biological predictors of medication treatment outcomes.^4,5,6,7,8,9,10^ However, EEG predictors identified in these studies are not robust enough to be used in clinical practice. For instance, 1 study^4^ found lower theta activity in responders to several selective serotonin reuptake inhibitor (SSRI) medications, whereas another study^5^ showed the opposite in responders to the SSRI fluoxetine. Responders to different SSRIs were also found to exhibit distinct patterns in alpha power.^6,7^ Failure to replicate across studies can be attributed to small sample size and the complexity and heterogeneity of depression. Testing individual EEG features in small, underpowered studies has hindered the development of EEG-based predictive models for clinical use.^11,12^ An alternative approach involves applying machine learning techniques on larger samples to potentially capture a more reliable neurobiological signature of response to antidepressants.^13,14,15^ However, despite some promising results, several challenges remain in implementing this approach.

The main challenge is the lack of generalizability of predictive models. Inadequate validation methods can lead to overfitting, especially with a small sample size.^16^ To avoid overfitting, proper internal and/or external validation is needed. Although internal validation is common due to scarce EEG data in depression studies,^13,15^ external validation is crucial from a clinical standpoint to establish model generalizability.^17^ However, to our knowledge, no study has rigorously used external validation with EEG-based models for predicting antidepressant response.^18^ Although some recent attempts have been made to validate such models with small independent cohorts of patients receiving different antidepressant modalities,^14^ independent replication is yet to be established.

Other challenges relate to the choice of EEG technology. Existing models have relied on high-density EEG devices with 64 or more channels, requiring extensive training and expertise to ensure data quality. Yet, for clinical viability, EEG signal must be easily recorded and robust to experimental setup noise. Developing models using features derived from portable EEG devices with fewer channels is an important step toward clinical EEG application. Efforts should be made to use fully automated pipelines for data cleaning and feature extraction, enabling nonexperts to use and process EEG data consistently across patients and sites, making them more suitable for clinical use.

To address these challenges, in this prognostic study we adapted the model developed by the Canadian Biomarker Integration Network in Depression (CAN-BIND)^13^ group to make it more suitable for a clinical environment and assessed its generalizability in an independent sample. Our adapted model was built using data from the CAN-BIND1 study,^19,20^ where participants received open-label escitalopram treatment across 6 clinical sites. Resting-state, eyes-closed EEG data were collected in 125 participants with high-density EEG devices. Zhdanov et al^13^ used features derived from 58 common electrodes across sites at baseline and week 2 to develop the previous model. In our study, only baseline data were used for clinical relevance, and a selection of 32 electrodes was made to emulate a setup closer to those used in a clinical environment with feasibility in mind. We extracted relative power density and multiscale entropy features and used a fully nested cross-validation on the CAN-BIND data set to obtain an unbiased estimation of the model’s performance. We aimed to achieve a higher accuracy compared with the mean accuracy of 56% reported in the 3 adequate-quality studies that were identified in a 2021 meta-analysis^18^ and focused on predicting response to medication treatments. Furthermore, we used an independent data set from the Establishing Moderators and Biosignatures of Antidepressant Response for Clinical Care (EMBARC) for Depression consortium,^21^ where 105 participants were randomized to receive sertraline treatment and 118 participants were randomized to receive placebo treatment across 4 clinical sites. We hypothesized that the predictive accuracy with external validation in the sertraline-treated EMBARC sample would be comparable to the predictive accuracy obtained with internal validation in the CAN-BIND sample.

## Methods

In this prognostic study, we developed and validated a multivariate model utilizing EEG data to predict response to SSRIs, adhering to the Transparent Reporting of a Multivariable Prediction Model for Individual Prognosis or Diagnosis (TRIPOD) reporting guideline.^22^ The CAN-BIND 1^19,20^ and EMBARC^21^ study protocols were approved by research ethics boards of participating institutions and complied with the Declaration of Helsinki.^23^ All participants provided written informed consent.

### Participants Sample

Only participants with baseline EEG data and who completed all assessments at week 8 were included in this study. In CAN-BIND,^19,20^ 6 Canadian sites recruited participants aged 18 to 60 years who met the *Diagnostic and Statistical Manual of Mental Disorders* (Fourth Edition) criteria for a major depressive episode of MDD. Participants received escitalopram during the first 8 weeks in an open-label design. The primary outcome was the Montgomery-Åsberg Depression Rating Scale (MADRS), and participants were identified as responders on the basis of a reduction in MADRS score of 50% or more from baseline to week 8.

In EMBARC,^21^ 4 US sites recruited participants aged 18 to 65 years with MDD according to the *Diagnostic and Statistical Manual of Mental Disorders* (Fourth Edition). Participants were randomized to receive sertraline or placebo during the first 8 weeks using a double-blind design. The primary outcome measure was the Hamilton Depression Rating Scale (HDRS-17), and participants with a reduction in their HDRS-17 score of 50% or more from baseline to week 8 were classified as responders. More details about CAN-BIND and EMBARC are given in eAppendix 1 in Supplement 1.

### EEG Data Recording and Processing

At baseline, EEG activity was recorded during the eyes-closed resting condition for participants at 4 sites in CAN-BIND^19,20^ and at all sites in EMBARC.^21^ Because EEG data were collected at multiple sites, data sets were standardized into a common site-independent format. A customized, fully automated cleaning pipeline was then used to remove artifacts from the data sets (more details in eAppendix 2, eTable 1, eAppendix 3, and eAppendix 4 in Supplement 1).

### EEG Features

#### Spectral Features

Power spectral density (absolute power) was estimated for each channel from 1 Hz to 50 Hz using the Welch method with 2-second nonoverlapping windows. Relative power was then obtained in 5 frequency bands—delta, theta, alpha, beta, and gamma—by calculating the ratio of the absolute power within a frequency band to the total sum of absolute power (1-50 Hz).

#### Multiscale Entropy Features

According to methods described in prior studies,^13,24,25^ multiscale entropy (MSE) was estimated for each channel on a scale of 1 to 70 using 30-second nonoverlapping windows. The following parameters were used for the computation: embedding dimension (*m* = 2) and similarity tolerance (*r* = 0.15). The MSE was then averaged within 3 timescale bands: fine timescales (1-20), medium timescales (21-35), and coarse timescales (36-70).

### EEG Features Reduction

To reduce the number of EEG features, the 32 electrodes were grouped into 14 brain regions, and features were averaged within each region. Asymmetry features were also computed by dividing features in the left hemisphere by features in the right hemisphere for 5 pairs of brain regions. This led to a total of 152 features. See more details on feature reduction in eAppendix 5 in Supplement 1 and information on electrode mapping in eFigure 1 in Supplement 1.

### Classifier Construction

For the current study, a nonparametric, supervised method called the *k*-nearest neighbors algorithm (kNN) was used. The kNN algorithm was chosen due to its capacity to find complex patterns, while making few assumptions regarding the data. With EEG signals exhibiting complex behavior with nonlinear dynamic properties, the kNN algorithm has been widely used in EEG research.^26,27,28,29^ This algorithm has only a few hyperparameters to be optimized (eg, number of neighbors *k* and distance metrics), which simplifies the training process. Finally, the kNN algorithm performs well in low-dimensional EEG data sets.^26^

### Assessments of Generalizability

#### Metrics

Balanced accuracy, sensitivity, and specificity were used to assess model predictive performance. The 95% CIs for these metrics were calculated using the normal approximation method or the Clopper-Pearson exact method, wherever appropriate. Responder status was considered as a positive outcome, and nonresponder as a negative outcome.

#### Internal Validation With CAN-BIND

A kNN classifier was trained using a nested leave-one-out cross-validation with the CAN-BIND data set.^30^ In this approach, no final model is built because a different set of optimal hyperparameters might be found in each fold. However, this procedure is essential to obtain an unbiased estimate of the model’s performance when no independent data set is available.^16^ Additionally, to reduce concerns related to overfitting, we conducted nested *k*-fold cross-validation with different values of *k* (more details in eAppendix 6 and eAppendix 7 in Supplement 1).^31^

#### External Validation With EMBARC

A kNN classifier was trained using a traditional leave-one-out cross-validation with the CAN-BIND data set. This process yielded a unique set of optimal hyperparameters, resulting in a final model capable of predicting new data on the basis of its k-nearest neighbors in the training set. The model’s generalizability was tested using the independent EMBARC sertraline group data set. Additionally, its ability to specifically predict response to SSRIs was evaluated by assessing its performance on the EMBARC placebo group data set.

### Feature Importance

To help in the interpretation of the model, permutation feature importance was used to identify the most impactful features. This procedure involved randomly permuting the values of each feature and measuring the resulting effect on the model’s performance. To ensure robustness, we repeated this procedure 100 times and computed the mean reduction in balanced accuracy as a measure of feature importance. We applied this approach to both internal validation with CAN-BIND and external validation with EMBARC (more details in eAppendix 8 in Supplement 1).

### Statistical Analysis

In this study, a machine learning approach was used instead of traditional statistical analysis. The ability of the kNN classifier to predict treatment outcomes from EEG data served as a statistical measure of difference between responders and nonresponders. All analyses were performed using MATLAB statistical software version R2020b (MathWorks) from January to December 2022. Statistical significance was set at 2-sided *P* < .05.

## Results

### Participant Characteristics

We analyzed data from 125 participants in CAN-BIND (mean [SD] age, 36.4 [13.0] years; 78 [62.4%] women) who had a mean (SD) MADRS score of 30.0 (5.8) at baseline (Table 1). We also analyzed data from 223 participants in EMBARC with 105 participants in sertraline group (mean [SD] age, 38.4 [13.8] years; 72 [68.6%] women) (Table 2) and 118 participants in the placebo group (mean [SD] age 37.3 [13.1] years; 73 [61.8%] women). Sertraline-treated participants had a mean (SD) HDRS score of 18.5 (4.4) at baseline.

**Table 1.** Demographics and Clinical Characteristics in the Canadian Biomarker Integration Network in Depression Cohort

Variable	Electroencephalography recording site					Responders, No. (%) (n = 56)	Nonresponders, No. (%) (n = 69)	*P* value
	CAMH (n = 7)	QNS (n = 18)	TGH (n = 46)	UBC (n = 54)	Total (N = 125)			
Age, mean (SD), y	30.4 (13.0)	42.7 (14.4)	35.8 (13.0)	35.6 (12.0)	36.4 (13.0)	36.1 (13.2)	36.7 (12.8)	.81
Sex, No. (%)								
Female	7 (100.0)	9 (50.0)	27 (58.7)	35 (64.8)	78 (62.4)	37 (66.1)	41 (59.4)	.45
Male	0	9 (50.0)	19 (41.3)	19 (35.2)	47 (37.6)	19 (33.9)	28 (40.6)	
MADRS wk 0 score, mean (SD)	28.0 (5.2)	30.0 (4.7)	32.4 (5.5)	28.3 (5.8)	30.0 (5.8)	29.4 (5.8)	30.5 (5.8)	.30
MADRS wk 8 score, mean (SD)	15.7 (6.0)	18.1 (10.2)	19.5 (11.9)	14.2 (9.2)	16.8 (10.5)	7.8 (5.0)	24.2 (7.6)	<.001
Change in MADRS score, mean (SD), %	44.5 (17.0)	39.2 (35.9)	39.6 (32.6)	49.8 (32.9)	44.2 (32.7)	73.5 (16.0)	20.5 (21.5)	<.001

Abbreviations: CAMH, Centre for Addiction and Mental Health; MADRS, Montgomery-Åsberg Depression Rating Scale; QNS, Queen’s University; TGH, Toronto General Hospital; UBC, University of British Columbia.

**Table 2.** Demographic and Clinical Characteristics in the Establishing Moderators and Biosignatures of Antidepressant Response for Clinical Care Sertraline Group

Variable	Electroencephalography recording site					Responders (n = 51)	Nonresponders (n = 54)	*P* value
	CU (n = 34)	MGH (n = 18)	TX (n = 36)	UM (n = 17)	Total (N = 105)			
Age, mean (SD), y	35.0 (11.9)	36.1 (15.1)	43.8 (13.5)	36.2 (14.4)	38.4 (13.8)	38.7 (13.1)	38.2 (14.5)	.86
Sex, No. (%)								
Female	24 (70.6)	10 (55.6)	27 (75.0)	11 (64.7)	72 (68.6)	35 (68.6)	37 (68.5)	.99
Male	10 (29.4)	8 (44.4)	9 (25.0)	6 (35.3)	33 (31.4)	16 (31.4)	17 (31.5)	
HDRS wk 0 score, mean (SD)	17.4 (4.3)	19.3 (4.1)	19.3 (4.9)	18.4 (3.9)	18.5 (4.4)	19.4 (4.2)	17.8 (4.6)	.07
HDRS wk 8 score, mean (SD)	7.4 (6.1)	12.6 (5.6)	12.4 (6.2)	10.9 (6.4)	10.6 (6.5)	5.3 (6.5)	15.5 (3.0)	<.001
Change in HDRS score, mean (SD), %	54.4 (39.4)	31.9 (33.4)	32.0 (38.3)	39.2 (35.4)	40.4 (38.3)	72.7 (38.3)	10.0 (27.4)	<.001

Abbreviations: CU, Columbia University; HDRS, Hamilton Depression Rating Scale; MGH, Massachusetts General Hospital; TX, University of Texas Southwestern Medical Center; UM, University of Michigan, Ann Arbor.

No differences were observed in age (36.4 years [95% CI, 34.1-38.7 years] vs 38.4 years [95% CI, 35.8-41.0 years]; *P* = .26) or sex (230 participants; χ^2^_1_ = 0.96; *P* = .33) between participants in CAN-BIND and sertraline-treated participants in EMBARC.

No differences were observed in age or sex between participants in CAN-BIND and sertraline-treated participants in EMBARC. However, converting HDRS scores to MADRS scores on the basis of item response theory–derived conversion tables^32,33^ revealed that sertraline-treated participants in EMBARC had significantly lower symptoms severity at baseline (mean [SD] HDRS converted to MADRS score, 25.5 [5.5]; 95% CI, 24.5-26.6) than participants in CAN-BIND (mean [SD] MADRS score, 30.0 [5.8]; 95% CI, 29.0-31.0; *P* < .01). See eAppendix 9, eFigure 2, and eTables 2-5 in Supplement 1 for more information on the CAN-BIND and EMBARC participants.

### Classifier Construction and Performance Estimation

#### Internal Validation With CAN-BIND

The kNN algorithm with nested leave-one-out cross-validation predicted response with a balanced accuracy of 64.2% (95% CI, 55.8%-72.6%), a sensitivity of 66.1% (95% CI, 53.7%-78.5%), and a specificity of 62.3% (95% CI, 50.1%-73.8%) in CAN-BIND (Table 3). In comparison, the nested 10-fold, cross-validation led to a balanced accuracy of 61.7% (95% CI, 52.1%-69.3%), a sensitivity of 62.3% (95% CI, 49.6%-75.0%), and a specificity of 59.1% (95% CI, 47.5%-70.7%). Results for other values of *k* in nested *k*-fold cross-validation can be found in eAppendix 10 and eFigure 4 in Supplement 1).

**Table 3.** Internal Validation With Canadian Biomarker Integration Network in Depression Data

Measure	Percentage (95% CI)				
	CAMH participants (n = 7)	QNS participants (n = 18)	TGH participants (n = 46)	UBC participants (n = 54)	Total participants (N = 125)
Sensitivity	66.7 (9.4-99.2)	100.0 (59.0-100.0)	65.0 (40.8-84.6)	57.7 (36.9-76.7)	66.1 (53.7-78.5)
Specificity	75.0 (19.4-99.4)	63.6 (30.8-89.1)	65.4 (44.3-82.8)	57.1 (37.2-75.5)	62.3 (50.1-73.8)
Balanced accuracy	70.8 (14.4-99.3)	81.8 (44.9-94.6)	65.2 (42.6-83.7)	57.4 (31.1-76.1)	64.2 (55.8-72.6)

Abbreviations: CAMH, Centre for Addiction and Mental Health; QNS, Queen’s University; TGH, Toronto General Hospital; UBC, University of British Columbia.

#### External Validation With EMBARC

After training the model with data from CAN-BIND, we assessed its ability to generalize to new data by testing it with data from the EMBARC sertraline-treated group (eAppendix 11 and eTable 8 in Supplement 1). The model achieved a balanced accuracy of 63.7% (95% CI, 54.5%-72.8%), a sensitivity of 58.8% (95% CI, 45.3%-72.3%), and specificity of 68.5% (95% CI, 56.1%-80.9%) (Table 4). Additionally, the model was tested with data from the EMBARC placebo group, but did not perform better than chance, yielding a balanced accuracy of 48.7% (95% CI, 39.3%-58.0%), a sensitivity of 50.0% (95% CI, 35.2%-64.8%), and specificity of 47.3% (95% CI, 35.9%-58.7%) (eTable 6 in Supplement 1). Furthermore, a significant difference was observed between the predictions in EMBARC sertraline and placebo groups (223 participants; χ^2^_1_ = 5.41; *P* = .02) (eTable 7 in Supplement 1). To assess the association of baseline symptoms severity differences between CAN-BIND and EMBARC participants with generalizability, we also conducted an exploratory analysis by excluding EMBARC participants with low baseline HDRS scores (eTable 9 in Supplement 1). More information regarding symptoms severity and generalizability can be found in eAppendix 12 and eFigure 5 in Supplement 1.

**Table 4.** External Validation With Establishing Moderators and Biosignatures of Antidepressant Response for Clinical Care Sertraline Group Data

Measure	Percentage (95% CI)				
	CU participants (n = 34)	MGH participants (n = 18)	TX participants (n = 36)	UM participants (n = 17)	Total participants (N = 105)
Sensitivity	61.9 (38.4-81.9)	80.0 (28.4-99.5)	52.9 (27.8-77.0)	50.0 (15.7-84.3)	58.8 (45.3-72.3)
Specificity	69.2 (38.6-90.9)	84.6 (54.6-98.1)	63.2 (38.4-83.7)	55.6 (21.2-86.3)	68.5 (56.1-80.9)
Balanced accuracy	65.6 (38.5-86.3)	82.3 (41.5-98.8)	58.1 (33.1-80.4)	52.8 (18.5-85.3)	63.7 (54.5-72.8)

Abbreviations: CU, Columbia University; MGH, Massachusetts General Hospital; TX, University of Texas Southwestern Medical Center; UM, University of Michigan Ann Arbor.

### Feature Importance

The process of randomly permuting the values of each of the 152 features resulted in a reduction in balanced accuracy ranging from 0.6% to 3.5% in internal validation with CAN-BIND, and from −0.2% to 3.7% in external validation with the EMBARC sertraline group (Figure). No clear structured pattern of informative features was identified when comparing internal and external validations. However, the asymmetry of power and multiscale entropy, especially in frontal, central, and parietal regions of the brain, provided the more robust features on the model’s performance in both validations. Feature importance for EMBARC placebo group can be found in eAppendix 13 and eFigure 6 in Supplement 1.

**Figure.**
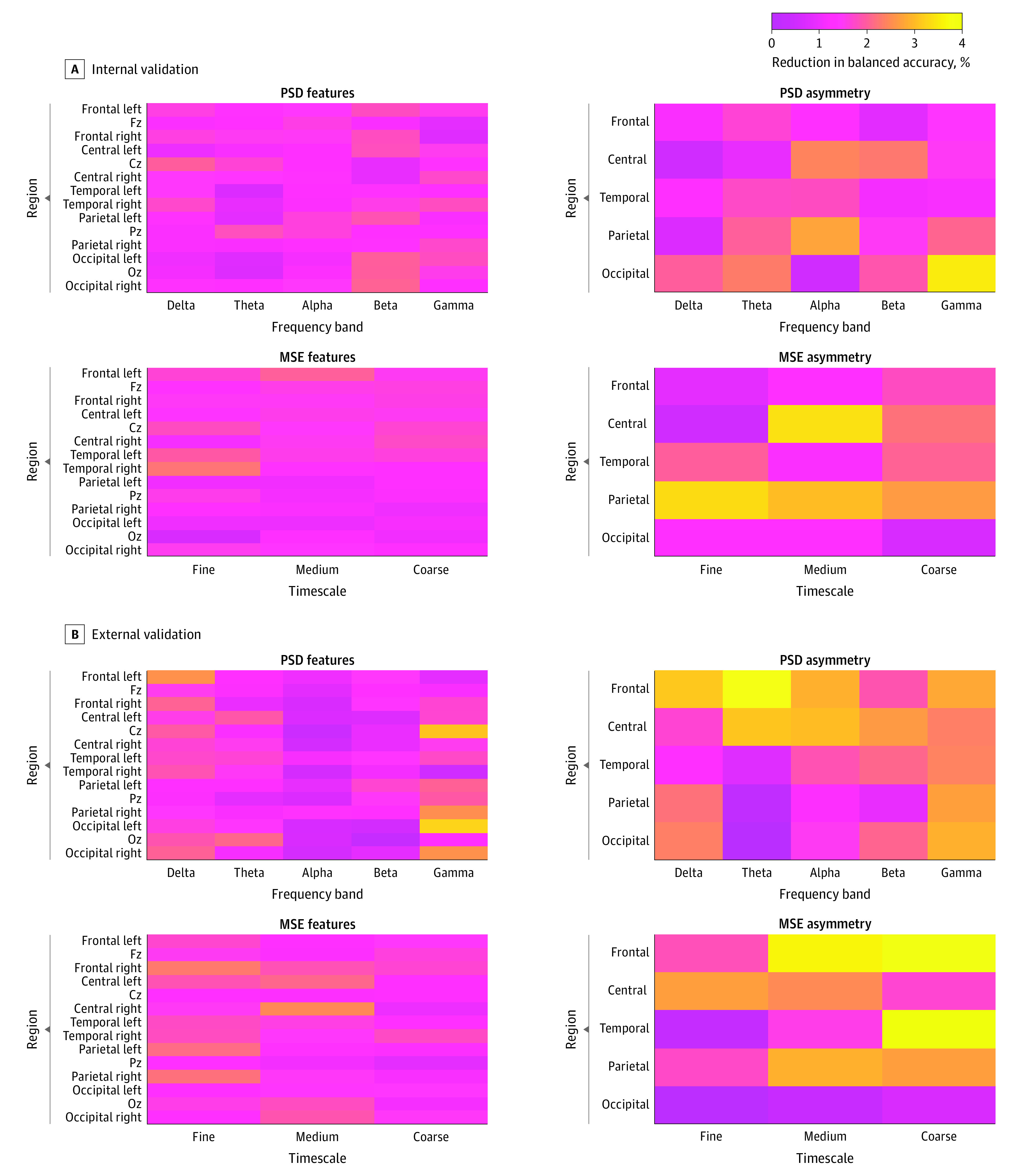
Feature Importance in Internal and External Validations The figure displays the feature importance results obtained in internal validation with the Canadian Biomarker Integration Network in Depression (CAN-BIND) cohort (A) and external validation with the Establishing Moderators and Biosignatures of Antidepressant Response for Clinical Care (EMBARC) sertraline-treated group (B), where each matrix corresponds to the importance of power spectral density (PSD), PSD asymmetry, multiscale entropy (MSE), and MSE asymmetry features. The depicted features in pink colors indicate a low level of importance for the model, whereas the features represented in yellow colors indicate a stronger level of importance. Feature importance was evaluated on the basis of the reduction in balanced accuracy in internal validation (A) and external validation (B). To enhance the clarity of the visualization, features exhibiting an increase in balanced accuracy were adjusted to 0%, indicating a lack of valuable information for predictive purposes (low level of importance). Cz indicates central zone; Fz, frontal zone; and Oz, occiptal zone.

## Discussion

In this prognostic study, we described an EEG-based model that predicts outcomes of treatment with SSRI antidepressants using 2 independent data sets. Our model achieved 64.2% balanced accuracy using internal validation with CAN-BIND, and 63.7% accuracy using external validation with EMBARC. This finding is of clinical importance because it provides a substantial improvement over trial-and-error methods. This observation is notably accentuated by the initial low response rates in the CAN-BIND and EMBARC samples. Accurately predicting treatment response in almost two-thirds of cases could lead to improved patient outcomes, reducing ineffective treatments and health care burden. Prior studies that reported higher accuracy with EEG-based models^13,34,35,36^ may have been overoptimistic due to limited sample sizes and overfitting in their validation procedures. In fact, Sajjadian et al^18^ found that of 54 studies predicting outcomes to medication treatment, only 8 met the criteria for adequate quality (ie, defined as having a minimum sample of 100 participants and an adequate validation method), and none of these studies used EEG for prediction. Moreover, the mean accuracy across these 8 adequate-quality studies was 63%, with prediction accuracies of 69% for treatment resistance, 60% for remission, and 56% for treatment response.^18^ Here, our study demonstrated that a model using EEG predictors for treatment response could perform as well as, if not better than, models using other types of predictors, such as genetic, clinical, or demographic data.

Furthermore, our EEG-based model presents substantial advantages for clinical practice. First, using objective measures such as EEG data to predict treatment response is preferable to relying on subjective subjective variables in clinical setting. Objective biomarkers have been suggested to reduce the self-stigma associated with seeking help and reporting symptoms on clinical questionnaires.^37^ Second, our model here generalized across 2 cohorts of patients who received different SSRIs (escitalopram and sertraline). For clinical practice in the near future, it will be probably more feasible to have a model that predicts response to a certain class of antidepressant (ie, SSRIs), than a predictive model for every single existing antidepressant medication, which would require extensive time and effort to collect sufficient EEG data. Furthermore, the model did not predict response to placebo treatment, suggesting its specificity for predicting outcomes with SSRIs and not just symptom improvement. Third, our model used only 32 electrodes, making it compatible with data recorded with portable EEG devices. Compared with research-grade EEG devices with 64 or more channels, these devices are more suitable for clinical settings with limited resources and time, prioritizing cost-effectiveness, ease of use, patient comfort, and signal quality over spatial resolution. Finally, the kNN algorithm used here is relatively simple and does not require complex training because it relies on observable data similarities to generate accurate predictions.

Regarding feature importance, the results suggest that power and multiscale entropy features from different regions are necessary to accurately predict treatment response. Interestingly, it was observed that asymmetry features in frontal, central, and parietal regions had the most impact on the model’s performance in both validations. This finding is consistent with previous studies that reported differences in EEG asymmetry features between responders and nonresponders to medications.^6,7,38,39^ These results support the potential usefulness of asymmetry measures in predicting treatment response and may contribute to the development of more accurate prediction models. Furthermore, the impact of individual features on the predictive model varied across the internal and external validation sets, indicating that a combination of features, rather than individual features, may be more relevant for accurate prediction. Overall, these findings highlight the importance of considering multiple features and their interactions when predicting treatment response to SSRIs.

An interesting point of this study was the association of symptom severity with model generalizability. The results suggest that the model might be better suited with participants experiencing moderate or severe depression. These findings highlight the potential need for distinct predictive models based on baseline symptom severity, driven by the inherent heterogeneity of depression.

### Limitations

This study has limitations regarding predictive accuracy. EEG-based models are not necessarily intended for stand-alone use in clinical practice, and future studies may enhance accuracy by incorporating other data sources. Recent research^40^ combining clinical, molecular, and magnetic resonance imaging data in the CAN-BIND sample showed improved treatment outcome prediction, but their models using baseline data achieved a mean balanced accuracy of 57%, which is still inferior to the accuracy obtained with EEG-alone here. Future studies could explore combining multimodal predictors with EEG to further improve predictive accuracy. It is also important to consider the theoretical ceiling in predicting treatment response in depression due to uncertainties associated with the response labels. For instance, Zhdanov and colleagues^13^ estimated the maximal achievable classification accuracy to be approximately 86%. Furthermore, EEG data can be sensitive to noise, artifacts, and other sources of variability, and the estimated 86% accuracy could be an upper limit, potentially attenuated in a clinical setting. Nonetheless, the achieved 64.2% balanced accuracy obtained in both internal and external validation suggests robustness of our EEG-based predictive model for treatment response in the presence of potential clinical variability and measurement errors. Other limitations include medication-free participants at baseline and exclusion of those with missing data. Future studies could use more diverse data sets including patients who are already taking an antidepressant or those for whom their first treatment failed to enhance the model’s generalizability and ensure its applicability to a wider range of patients. Additionally, validating the findings in larger cohorts through future research or follow-up studies would be valuable for further refining the predictive model’s performance. Furthermore, the study focused only on eyes-closed resting-state EEG databased on previous evidence.^13^ However, Wu et al^14^ found eyes-open condition to be more predictive of sertraline response. Future research may wish to further explore the value of both types of EEG data to improve predictive accuracy.

## Conclusions

We developed an EEG-based model with potential for clinical use to predict response to SSRIs, taking into account the practical application of EEG technology in a clinical setting. This study is the first, to our knowledge, to present replicable evidence of such a model and be validated in a large, independent cohort. These findings represent a substantial advancement toward using EEG in future clinical practice and supporting its potential to match patients with MDD to optimized treatment.

## Notes

Supplement 1. **eAppendix 1.** Participant Samples **eAppendix 2.** EEG Data Recording **eTable 1.** EEG Amplifier Settings Used in CAN-BIND and EMBARC **eAppendix 3.** EEG Data Standardization **eAppendix 4.** EEG Data Processing **eAppendix 5.** EEG Features Reduction **eFigure 1.** Division of the Brain Regions and Their Included Electrodes **eAppendix 6.** Nested Leave-One-Out Cross-Validation **eAppendix 7.** Nested K-Fold Cross-Validation **eAppendix 8.** Feature Importance **eAppendix 9.** Results: Participant Characteristics **eFigure 2.** Flow of Participants in CAN-BIND **eTable 2.** Demographics and Clinical Characteristics in CAN-BIND of Participants Who Completed Week 8 **eTable 3.** Demographics and Clinical Characteristics in CAN-BIND of Participants Who Completed Week 8 and Had EEG Recording **eFigure 3.** Flow of Participants in EMBARC **eTable 4.** Demographics and Clinical Characteristics in EMBARC Active Arm of Participants Who Completed Week 8 **eTable 5.** Demographics and Clinical Characteristics in EMBARC Active Arm of Participants Who Completed Week 8 and Had EEG Recording **eAppendix 10.** Classifier Construction and Performance Estimation: Internal Validation with CAN-BIND **eFigure 4.** Balanced Accuracy in Internal Validation with CAN-BIND **eAppendix 11.** Classifier Construction and Performance Estimation: External Validation with EMBARC **eTable 6.** External Validation With EMBARC Placebo Arm **eTable 7.** Contingency Table With Correct and Incorrect Predictions in EMBARC Sertraline and Placebo Arms **eAppendix 12.** Impact of Symptoms Severity on Generalizability **eTable 8.** External Validation With EMBARC Sertraline Arm (Participants With HDRS ≥16) **eTable 9.** External Validation With EMBARC Placebo Arm (Participants With HDRS ≥16) **eFigure 5.** Impact of Symptoms Severity on Generalizability **eAppendix 13.** Feature Importance **eFigure 6.** Feature Importance in External Validation With EMBARC Placebo Arm
eReferences

Supplement 2.
Data Sharing Statement
