# Supplementary material for: Developing an Electroencephalography-Based Model for Predicting Response to Antidepressant Medication
Source: JAMA Netw Open. 2023 Sep 28;6(9):e2336094. doi: 10.1001/jamanetworkopen.2023.36094 (PMC10539986; doi:10.1001/jamanetworkopen.2023.36094)
Supplement: Supplement 1. — eAppendix 1. Participant Samples eAppendix 2. EEG Data Recording eTable 1. EEG Amplifier Settings Used in CAN-BIND and EMBARC eAppendix 3. EEG Data Standardization eAppendix 4. EEG Data Processing eAppendix 5. EEG Features Reduction eFigure 1. Division of the Brain Regions and Their Included Electrodes eAppendix 6. Nested Leave-One-Out Cross-Validation eAppendix 7. Nested K-Fold Cross-Validation eAppendix 8. Feature Importance eAppendix 9. Results: Participant Characteristics eFigure 2. Flow of Participants in CAN-BIND eTable 2. Demographics and Clinical Characteristics in CAN-BIND of Participants Who Completed Week 8 eTable 3. Demographics and Clinical Characteristics in CAN-BIND of Participants Who Completed Week 8 and Had EEG Recording eFigure 3. Flow of Participants in EMBARC eTable 4. Demographics and Clinical Characteristics in EMBARC Active Arm of Participants Who Completed Week 8 eTable 5. Demographics and Clinical Characteristics in EMBARC Active Arm of Participants Who Completed Week 8 and Had EEG Recording eAppendix 10. Classifier Construction and Performance Estimation: Internal Validation with CAN-BIND eFigure 4. Balanced Accuracy in Internal Validation with CAN-BIND eAppendix 11. Classifier Construction and Performance Estimation: External Validation with EMBARC eTable 6. External Validation With EMBARC Placebo Arm eTable 7. Contingency Table With Correct and Incorrect Predictions in EMBARC Sertraline and Placebo Arms eAppendix 12. Impact of Symptoms Severity on Generalizability eTable 8. External Validation With EMBARC Sertraline Arm (Participants With HDRS ≥16) eTable 9. External Validation With EMBARC Placebo Arm (Participants With HDRS ≥16) eFigure 5. Impact of Symptoms Severity on Generalizability eAppendix 13. Feature Importance eFigure 6. Feature Importance in External Validation With EMBARC Placebo Arm eReferences [file jamanetwopen-e2336094-s001.pdf]

Supplemental Online Content

Schwartzmann B, Dhami P, Uher R, et al. Developing an electroencephalography-based model for predicting response to antidepressant medication. *JAMA Netw Open*. 2023;6(9):e2336094. doi:10.1001/jamanetworkopen.2023.36094

eAppendix 1. Participant Samples

eAppendix 2. EEG Data Recording

eTable 1. EEG Amplifier Settings Used in CAN-BIND and EMBARC

eAppendix 3. EEG Data Standardization

eAppendix 4. EEG Data Processing

eAppendix 5. EEG Features Reduction

eFigure 1. Division of the Brain Regions and Their Included Electrodes

eAppendix 6. Nested Leave-One-Out Cross-Validation

eAppendix 7. Nested K-Fold Cross-Validation

eAppendix 8. Feature Importance

eAppendix 9. Results: Participant Characteristics

eFigure 2. Flow of Participants in CAN-BIND

eTable 2. Demographics and Clinical Characteristics in CAN-BIND of Participants Who Completed Week 8

eTable 3. Demographics and Clinical Characteristics in CAN-BIND of Participants Who Completed Week 8 and Had EEG Recording

eFigure 3. Flow of Participants in EMBARC

eTable 4. Demographics and Clinical Characteristics in EMBARC Active Arm of Participants Who Completed Week 8

eTable 5. Demographics and Clinical Characteristics in EMBARC Active Arm of Participants Who Completed Week 8 and Had EEG Recording

eAppendix 10. Classifier Construction and Performance Estimation: Internal Validation with CAN-BIND

eFigure 4. Balanced Accuracy in Internal Validation with CAN-BIND

eAppendix 11. Classifier Construction and Performance Estimation: External Validation with EMBARC

eTable 6. External Validation With EMBARC Placebo Arm

© 2023 Schwartzmann B et al. *JAMA Network Open.*

eTable 7. Contingency Table With Correct and Incorrect Predictions in EMBARC Sertraline and Placebo Arms

eAppendix 12. Impact of Symptoms Severity on Generalizability

eTable 8. External Validation With EMBARC Sertraline Arm (Participants With HDRS >=16)

eTable 9. External Validation With EMBARC Placebo Arm (Participants With HDRS >=16)

eFigure 5. Impact of Symptoms Severity on Generalizability

eAppendix 13. Feature Importance

eFigure 6. Feature Importance in External Validation With EMBARC Placebo Arm

eReferences

This supplemental material has been provided by the authors to give readers additional information about their work.

© 2023 Schwartzmann B et al. *JAMA Network Open.*

eAppendix 1. Participant Samples

CAN-BIND: In the CAN-BIND 1 study [1,2], participants were required at six sites in Canada: University of Calgary (UCA), University of British Columbia (UBC), McMaster University (MAC), Queen's University (QNS), Toronto General Hospital (TGH), and Centre for Addiction and Mental Health (CAMH). All participants provided informed written consent. The study protocol was approved by Research Ethics Boards of participating institutions. It was in accordance with the Declaration of Helsinki and was registered with clinicaltrials.gov (https://clinicaltrials.gov/ct2/show/NCT01655706).

Eligibility: Participants were required to be between 18 to 60 years old and meet the following inclusion criteria: (1) meet Diagnostic and Statistical Manual of Mental Disorders (DSM-IV-TR) criteria for a Major Depressive Episode in Major Depressive Disorder by the Mini International Neuropsychiatric Interview (MINI); (2) episode duration of at least 3 months; (3) free of psychotropic medications for at least 5 half-lives before baseline visit (4) Montgomery–Åsberg Depression Rating Scale (MADRS) score ≥ 24; (5) fluency in English. Study exclusion criteria included: (1) any Axis I diagnosis other than MDD that is considered the primary diagnosis; (2) Bipolar I or Bipolar II diagnosis; (3) presence of a significant Axis II diagnosis (borderline, antisocial); (4) high suicidal risk, defined by clinician judgment; (5) substance dependence/abuse in the past 6 months; (6) presence of significant neurological disorders, head trauma or other unstable medical conditions; (7) pregnant or breastfeeding; (8) failure of 3 or more adequate pharmacologic interventions (as determined by the Antidepressant Treatment History Form); (9) started psychological treatment within the past 3 months with the intent of continuing treatment; (10) patients who have previously failed escitalopram or showed intolerance to escitalopram and patients at risk for hypomanic switch (i.e. with a history of antidepressant hypomania).

Treatment: During this 16-week trial, participants received escitalopram 10-20 mg during the first 8 weeks in an open-label design. The dose could be reduced back to 10 mg if not tolerated. At week 8, participants were identified as responders to escitalopram based on a reduction in MADRS score of ≥ 50% from baseline to week 8. This group continued to receive escitalopram for the remaining 8 weeks, while non-responders at week 8 received aripiprazole (2-10 mg daily) as an add-on treatment to escitalopram. The primary outcome measure was change in MADRS, from baseline to 8 and 16 weeks.

EMBARC: In the EMBARC study [3], four sites in USA recruited participants: Massachusetts General (MG) Hospital, University of Michigan (UM) Ann Arbor, Columbia University (CU), University of Texas (TX) Southwestern Medical Center. Written informed consents were obtained for all participants. The study protocol was approved by Research Ethics Boards of participating institutions. It was in accordance with the Declaration of Helsinki and was registered with clinicaltrials.gov (https://clinicaltrials.gov/ct2/show/NCT01407094).

Eligibility: Participants had to between 18 to 65 years to be enrolled in the study. Inclusion criteria were: (1) outpatients with a current primary diagnosis of nonpsychotic recurrent or chronic MDD per the Structured Clinical Interview for DSM Disorders (SCID-I); (2) Quick Inventory of Depressive Symptomatology - Self Report (QIDS-SR) score of 14 or higher at screening visit and randomization (baseline) visit; (3) no failed antidepressant trials at adequate dose and duration, as defined by the Massachusetts General Hospital Antidepressant Treatment Response Questionnaire (MGH-ATRQ), in the current episode; (4) agrees to, and is eligible for, all biomarker procedures (EEG/psychological testing, magnetic resonance imaging (MRI), and blood draws). Study exclusion criteria included: (1) history of inadequate response (to trials at adequate dose for adequate duration) or poor tolerability to sertraline (SERT) or bupropion

© 2023 Schwartzmann B et al. *JAMA Network Open.*

(BUP); (2) pregnant or breastfeeding; (3) plan to become pregnant over the ensuing 12 months following study entry or are sexually active and not using adequate contraception; (4) history (lifetime) of psychotic depression, schizophrenia, bipolar (I, II, or NOS) disorder, schizoaffective disorder, or other; (5) Axis I psychotic disorder; (6) current primary anxiety disorder diagnosis; (7) meeting DSM-IV criteria for substance abuse in the last 2 months or substance dependence in the last 6 months (except for nicotine); (8) require immediate hospitalization for psychiatric disorder; (9) have an unstable general medical condition (GMC) that will likely require hospitalization or to be deemed terminal (life expectancy < 6 months after study entry); (10) require medications for their GMCs that contraindicate any study medication; (11) have epilepsy or other conditions requiring an anticonvulsant; (12) receiving or have received during the index episode vagus nerve stimulation, ECT, or rTMS, or other somatic antidepressant treatments; (13) currently taking any of the following exclusionary medications: antipsychotic medications, anticonvulsant medications, mood stabilizers, central nervous system stimulants, daily use of benzodiazepines or hypnotics, or antidepressant medication used for the treatment of depression or other purposes such as smoking cessation, since these agents may interfere with the testing of the major hypotheses under study. Non excluded concomitant medications are acceptable as long as their clinician determines that antidepressant treatment is safe and appropriate; (14) significant liver disease that would contraindicate any study medication; (15) taking thyroid medication for hypothyroidism may be included only if they have been stable on the thyroid medication for 3 months; (16) using agents that are potential augmenting agents (e.g., T3 in the absence of thyroid disease, SAMe, St. John's Wort, lithium, buspirone, Omega 3 fatty acids); (17) therapy that is depression specific, such as CBT or Interpersonal Psychotherapy of Depression (IPT) is not allowed during participation (participants can participate if they are receiving psychotherapy that

© 2023 Schwartzmann B et al. *JAMA Network Open.*

is not targeting the symptoms of depression, such as supportive therapy, marital therapy); (18) currently actively suicidal or considered a high suicide risk; (19) are currently enrolled in another study, and participation in that study contraindicates participation in the EMBARC study; (20) any reason not listed herein yet, determined by the site PI, medical personnel, or designee that constitutes good clinical practice and that would in the opinion of the site PI, medical personnel, or designee make participation in the study hazardous.

Treatment: Participants were randomized to an 8-week course of sertraline or placebo using a double-blind design. Sertraline was started at 50 mg daily and could be increased to 200 mg daily for non-responders. The same dosing schedule was applied to placebo treatment. Using the Clinical Global Improvement scale (CGI), participants were identified as responders (>= 50% improvement on the CGI from baseline to week 8) to their respective treatment at week 8. Responding participants continued to receive either sertraline or placebo for a further 8 weeks; non-responding participants to sertraline at week 8 received an augmented treatment with bupropion (150-450 mg daily), and non-responding participants to placebo were switched to sertraline. The primary outcome measure was the Hamilton Depression Rating Scale (HDRS-17) and was assessed every week during the trial.

eAppendix 2. EEG Data Recording

CAN-BIND: EEG data were collected at 4 sites (CAMH, TGH, QNS and UBC). Eight minutes of EEG activity were recorded from all participants during the eyes-closed resting condition at baseline.

EMBARC: All 4 sites recorded resting-state EEG during four 2-min blocks (two blocks for eyes-open and two blocks for eye-closed) at baseline.

© 2023 Schwartzmann B et al. *JAMA Network Open.*

The EEG amplifier settings across sites in the CAN-BIND and EMBARC studies are summarized in e**Table 1**.

eTable 1. EEG Amplifier Settings Used in CAN-BIND and EMBARC

	CAN-BIND				EMBARC			
	**CAMH**	**QNS**	**TGH**	**UBC**	**CU**	**MG**	**TX**	**UM**
**Amplifier**	NeuroScan Synamp	Geodesic Net	Biosemi	QuickAmp	Biosemi	Geodesic Net	NeuroScan Synamp	NeuroScan Synamp
**Number of electrodes**	64 + 4 EOG	128	64 + 4 EOG	64 + 4 EOG	72	128	62	60
**Montage**	10/10	HydroCel GSN 128	10/10	10/10	10/10	HydroCel GSN 128	10/10	10/10
**Sampling rate**	1000Hz	1000Hz	512Hz	1000Hz	256Hz	250Hz	250Hz	250Hz
**Reference**	Posterior to Cz	Cz	CMS/DRL	Cz	CMS/DRL	Cz	Nose	Nose
**Filtering**	0.05-100Hz	0.04-100Hz	DC -3dB attenuation at 102.4Hz	0.01-499Hz	DC -3dB attenuation at 51.2Hz	0.01-100Hz	0-100Hz	0.5-100Hz

CAMH, Centre for Addiction and Mental Health; QNS, Queen's University; TGH, Toronto General Hospital; UBC, University of British Columbia; MCU, McMaster University; UCA, University of Calgary; CU, Columbia University; MG, Massachusetts General Hospital; UM, University of Michigan Ann Arbor; TX, University of Texas Southwestern Medical Center.

eAppendix 3. EEG Data Standardization

CAN-BIND: EEG data were standardized according to the following processing steps: selection of 32 common electrodes, resampling to 500Hz, re-referencing to the average reference. In addition, only the first 5-minutes of the recording in the eyes-closed conditions was used. The 32 electrodes, 500Hz sampling rate, and 5-minutes duration data were chosen to match the new EEG device and recording procedures that will be used in future studies at CAN-BIND.

© 2023 Schwartzmann B et al. *JAMA Network Open.*

EMBARC: A similar standardization was performed, except for the sampling rate set to 250Hz (see **eTable 1**). The duration of the recording also slightly differed, as only a total of 4-minutes with the eye-closed condition was recorded in the EMBARC study.

eAppendix 4. EEG Data Processing

A customized, fully automated pipeline was used to remove artifacts from the data. This pipeline was build using EEGLAB plugin functions and an adapted ERPEEG toolbox [4]. If needed, data were first resampled to the right sampling rate. A second-order Butterworth IIR filter was then applied to high-pass filter data at 1 Hz. In a third step, bad channels were deleted using the EEGLAB plugin clean_rawdata, and dad segments are corrected using Artifact Subspace Reconstruction algorithm [5]. ZapLine method was then employed to eliminated power line artifacts [6]. Independent component analysis was conducted to decompose data into independent components in step five. Components associated with eye movement, eyes blinks, muscle, and cardiac artifacts were then removed using the ICLabel algorithm [7]. Finally, channels previously deleted were then reconstructed with spherical interpolation, and data were re-referenced to average.

eAppendix 5. EEG Features Reduction

To reduce the number of EEG features, the 32 electrodes were grouped into 14 brain regions (bilateral frontal, temporal, central, parietal, and occipital regions, and midline frontal, central, parietal and occipital regions, **eFigure 1**) and features were averaged within each region. Asymmetry features were also computed by dividing features in the left hemisphere by features in the right hemisphere for 5 pairs of brain regions. In total, we had 152 features: 95 spectral

© 2023 Schwartzmann B et al. *JAMA Network Open.*

features (5 frequency bands x 14 brain regions + 5 frequency bands x 5 brain region pairs) and 57 multiscale entropy features (3 timescale bands x 14 brain regions + 3 timescale bands x 5 brain region pairs).

eFigure 1. Division of the Brain Regions and Their Included Electrodes

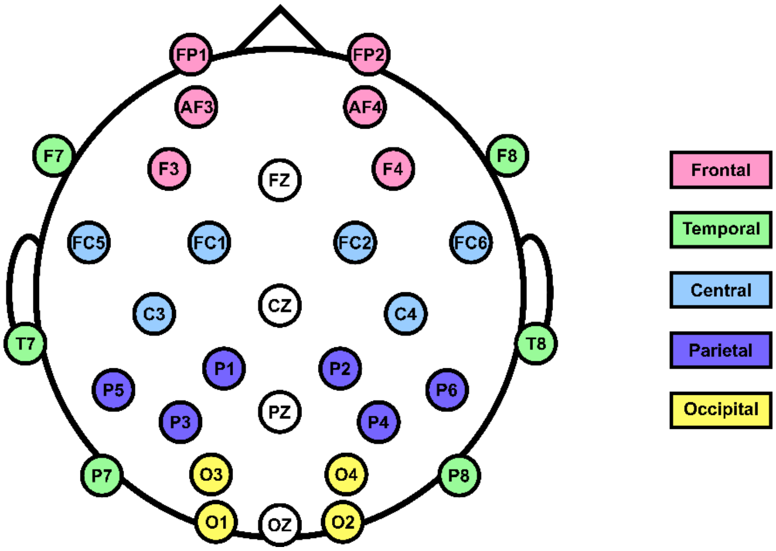

Frontal left (FP1, AF3 and F3), Frontal midline (FZ), Frontal right (FP2, AF4 and F4), Temporal left (F7, T7 and P7), Temporal right (F8, T8, P8), Central left (FC5, FC1 and C3), Central midline (CZ), Central right (FC2, FC6, C6), Parietal left (P1, P3 and P5), Parietal midline (PZ), Parietal right (P2, P4 and P6), Occipital left (O1 and O3), Occipital midline (OZ) and Occipital right (O2 and O4).

eAppendix 6. Nested Leave-One-Out Cross-Validation

With limited sample size, leave-one-out cross-validation is often used to evaluate the performance of a classifier [8]. This strategy allows more training samples to be used in each iteration and reduces the variability of model performance estimations due to the random partition mechanism in k-fold cross validation [8]. With nested leave-one-out cross-validation, the outer loop contains n folds (where n corresponds to the number of samples in the dataset), and the inner loop contains n-1 folds. Using the training part of each fold in the outer loop,

© 2023 Schwartzmann B et al. *JAMA Network Open.*

hyperparameters are tuned in the inner loop. This ensure that the test part of each fold in the outer loop is not used for optimization of the hyperparameters. In this approach, no final model is built as a different set of optimal hyperparameters might be obtained in each fold. However, this procedure is necessary to get an unbiased estimate of the model performance when no independent dataset is available [9].

eAppendix 7. Nested K-Fold Cross-Validation

To reduce concerns related to overfitting, we conducted a comparison between nested leave-one-out cross-validation and nested k-fold cross-validation [10]. In the outer loop, we experimented with different values of k, such as 5, 10, 20, 30, and 40. Similarly, the inner loop comprised k-fold iterations, matching the number chosen for k in each case. To address the potential variability introduced by the random partitioning in k-fold cross-validation, we repeated the entire process 10 times. This approach aimed to reduce the impact of chance variations and provide more robust estimations of model performance.

eAppendix 8. Feature Importance

Feature importance was assessed using random permutation in both interval validation and external validation. For internal validation using nested leave-one-out cross-validation with CAN-BIND, the feature values in the testing fold were randomly permuted while keeping the training fold intact. The model was then trained on the training fold and evaluated on the testing fold with permuted values. For each feature, the balanced accuracy was computed and compared to the balanced accuracy obtained with the original data. For external validation with EMBARC, the same process was used, where the feature values in the testing set (in our case, EMBARC dataset) were randomly permuted while keeping the training set intact (in our case, CAN-BIND

dataset). In both internal and external validations, this procedure was repeated 100 times and we computed the average reduction in balanced accuracy as a measure of feature importance.

eAppendix 9. Results: Participant Characteristics

CAN-BIND. Of 211 participants recruited in the CAN-BIND 1 study, 192 completed the baseline visit and received at least one dose of escitalopram. Out of these 192 participants, 180 completed all measures up to week 8. In this study, we analyzed data from 125 participants who were recruited at sites where EEG data were collected (**eFigure 2**). Demographic and clinical characteristics of participants are summarized in **eTables 2 and 3**.

EMBARC. Of 634 participants enrolled in the EMBARC study [3], 296 entered the randomization phase and 287 received at least one dose of sertraline or placebo. Out of these 287 participants, 240 completed all measures up to week 8, with 114 in the active arm (sertraline-treated) and 126 in the placebo arm. At baseline, EEG data from seventeen participants were missing or unusable, leaving a total 105 participants in the active arm and 118 participants in the placebo arm for the analysis (**eFigure 3**). Demographic and clinical characteristics for the participants in the active arm are summarized in **eTables 4 and 5**.

© 2023 Schwartzmann B et al. *JAMA Network Open.*

eFigure 2. Flow of Participants in CAN-BIND

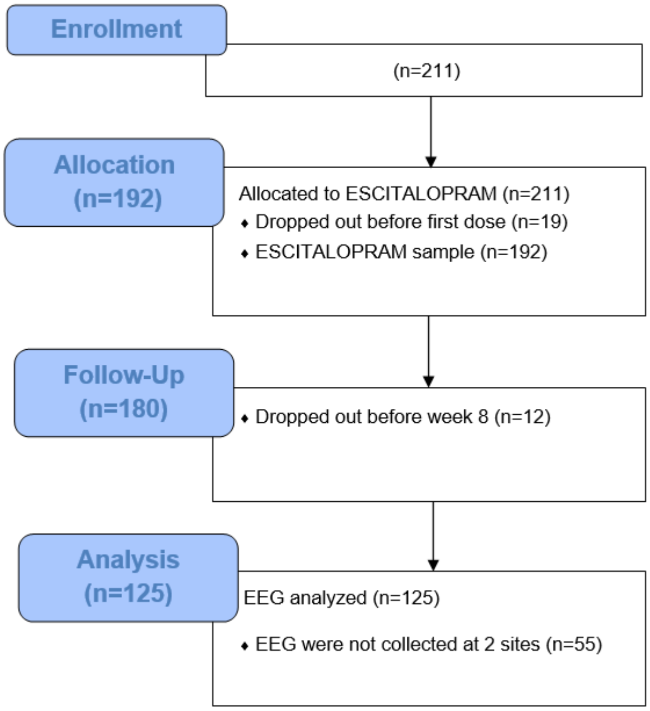

eTable 2. Demographics and Clinical Characteristics in CAN-BIND of Participants Who Completed Week 8

Variable	CAMH (n = 7)	QNS (n = 18)	TGH (n = 47)	UBC (n = 54)	MUC (n = 28)	UCA (n = 26)	ALL (n = 180)	Responders (n = 85)	Non-responders (n = 95)	P value
Age, mean (SD), y	30.4 (13.0)	42.7 (14.4)	35.7 (12.9)	35.6 (12.0)	34.0 (12.6)	32.5 (11.5)	35.6 (12.7)	35.0 (12.2)	35.8 (13.2)	0.68
Female	7 (100)	9 (50)	28 (59.6)	35 (64.8)	20 (71.4)	13 (50.0)	112 (62.2)	55 (64.7)	57 (60.0)	0.52
MADRS Week 0, mean (SD)	28.0 (5.2)	30.0 (4.7)	32.2 (5.6)	28.3 (5.8)	29.2 (5.3)	30.9 (4.4)	30.0 (5.5)	29.5 (5.6)	30.5 (5.5)	0.24
MADRS Week 8, mean (SD)	15.7 (6.0)	18.1 (10.2)	19.2 (12.0)	14.2 (9.2)	14.6 (8.9)	15.9 (9.2)	16.3 (10.1)	8.08 (5.0)	23.7 (7.3)	< 0.001
% Change in MADRS, mean (SD)	44.5 (17.0)	39.2 (35.9)	40.6 (33.0)	49.8 (32.9)	50.0 (29.5)	49.1 (29.5)	46.1 (31.7)	73.1 (15.7)	21.9 (21.1)	< 0.001

CAMH, Centre for Addiction and Mental Health; QNS, Queen's University; TGH, Toronto General Hospital; UBC, University of British Columbia; MCU, McMaster University; UCA, University of Calgary; MADRS, Montgomery-Åsberg Depression Rating Scale. Note that sites MCU and UCA did not conduct EEG recordings.

© 2023 Schwartzmann B et al. *JAMA Network Open.*

eTable 3. Demographics and Clinical Characteristics in CAN-BIND of Participants Who Completed Week 8 and Had EEG Recording

Variable	CAMH (n = 7)	QNS (n = 18)	TGH (n = 46)	UBC (n = 54)	ALL (n = 125)	Responders (n = 56)	Non-responders (n = 69)	P value
Age, mean (SD), y	30.4 (13.0)	42.7 (14.4)	35.8 (13.0)	35.6 (12.0)	36.4 (13.0)	36.1 (13.2)	36.7 (12.8)	0.81
Female	7 (100)	9 (50)	27 (58.7)	35 (64.8)	78 (62.4)	37 (66.1)	41 (59.4)	0.45
MADRS Week 0, mean (SD)	28.0 (5.2)	30.0 (4.7)	32.4 (5.5)	28.3 (5.8)	30.0 (5.8)	29.4 (5.8)	30.5 (5.8)	0.30
MADRS Week 8, mean (SD)	15.7 (6.0)	18.1 (10.2)	19.5 (11.9)	14.2 (9.2)	16.8 (10.5)	7.8 (5.0)	24.2 (7.6)	< 0.001
% Change in MADRS, mean (SD)	44.5 (17.0)	39.2 (35.9)	39.6 (32.6)	49.8 (32.9)	44.2 (32.7)	73.5 (16.0)	20.5 (21.5)	< 0.001

CAMH, Centre for Addiction and Mental Health; QNS, Queen's University; TGH, Toronto General Hospital; UBC, University of British Columbia; MADRS, Montgomery-Åsberg Depression Rating Scale.

eFigure 3. Flow of Participants in EMBARC

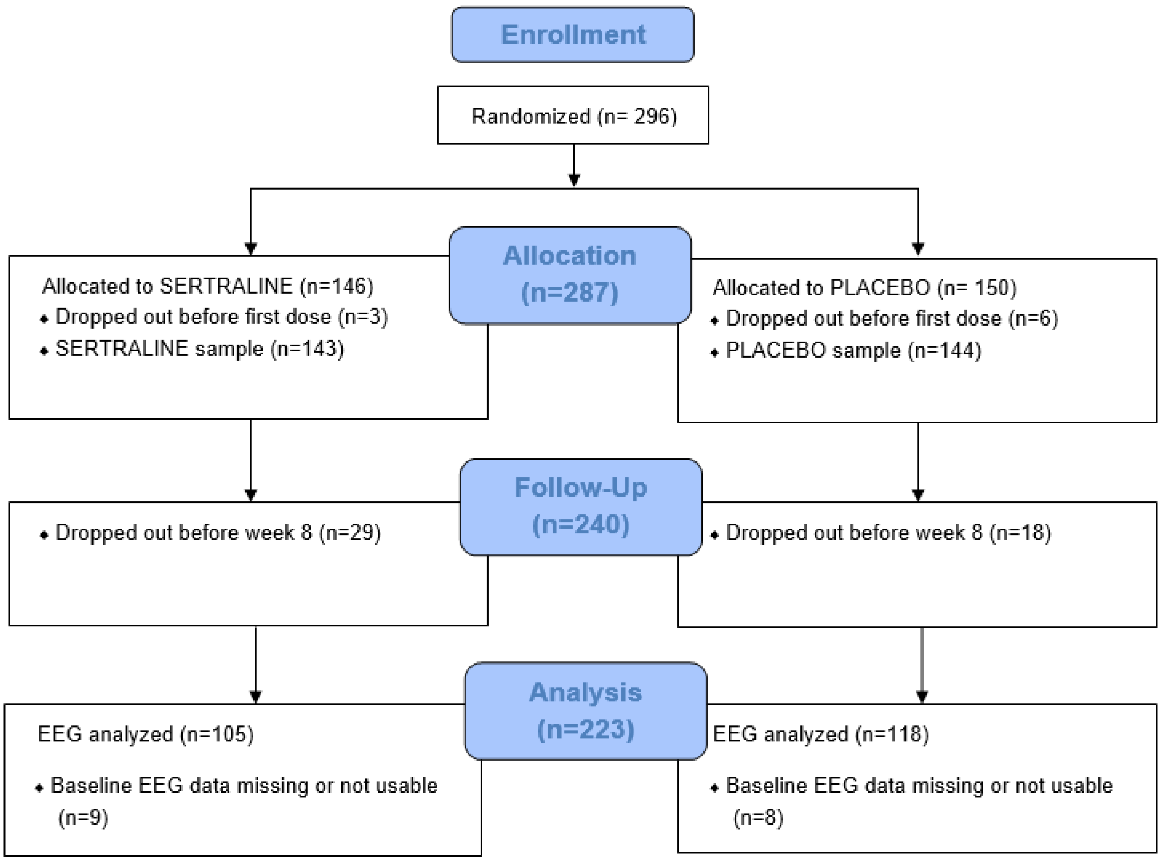

© 2023 Schwartzmann B et al. *JAMA Network Open.*

eTable 4. Demographics and Clinical Characteristics in EMBARC Active Arm of Participants Who Completed Week 8

Variable	CU (n = 37)	MG (n = 20)	TX (n = 38)	UM (n = 19)	ALL (n = 114)	Responders (n = 54)	Non-responders (n = 60)	P value
Age, mean (SD), y	34.9 (12.2)	34.9 (14.8)	44.0 (13.6)	35.6 (14.3)	38.1 (14.0)	39.2 (13.4)	37.0 (14.5)	0.46
Female	26 (70.3)	12 (60.0)	28 (73.7)	11 (57.9)	77 (67.5)	36 (66.7)	41 (68.3)	0.85
HDRS Week 0, mean (SD)	17.7 (4.3)	19.0 (4.0)	19.2 (4.8)	18.9 (4.1)	18.6 (4.4)	19.3 (4.1)	18.0 (4.6)	0.13
HDRS Week 8, mean (SD)	7.5 (6.0)	12.5 (5.5)	12.3 (6.3)	11.9 (7.1)	10.7 (6.5)	5.3 (3.0)	15.6 (4.8)	< 0.001
% Change in HDRS, mean (SD)	54.5 (38.1)	31.4 (32.0)	32.7 (38.3)	36.6 (34.8)	40.2 (37.5)	72.5 (13.6)	11.1 (26.7)	< 0.001

CU, Columbia University; MG, Massachusetts General Hospital; UM, University of Michigan Ann Arbor; TX, University of Texas Southwestern Medical Center; HDRS, Hamilton Depression Rating Scale.

eTable 5. Demographics and Clinical Characteristics in EMBARC Active Arm of Participants Who Completed Week 8 and Had EEG Recording

Variable	CU (n = 34)	MG (n = 18)	TX (n = 36)	UM (n = 17)	ALL (n = 105)	Responders (n = 51)	Non-responders (n = 54)	P value
Age, mean (SD), y	35.0 (11.9)	36.1 (15.1)	43.8 (13.5)	36.2 (14.4)	38.4 (13.8)	38.7 (13.1)	38.2 (14.5)	0.86
Female	24 (70.6)	10 (55.6)	27 (75.0)	11 (64.7)	72 (68.6)	35 (68.6)	37 (68.5)	0.99
HDRS Week 0, mean (SD)	17.4 (4.3)	19.3 (4.1)	19.3 (4.9)	18.4 (3.9)	18.5 (4.4)	19.4 (4.2)	17.8 (4.6)	0.07
HDRS Week 8, mean (SD)	7.4 (6.1)	12.6 (5.6)	12.4 (6.2)	10.9 (6.4)	10.6 (6.5)	5.3 (6.5)	15.5 (3.0)	< 0.001
% Change in HDRS, mean (SD)	54.4 (39.4)	31.9 (33.4)	32.0 (38.3)	39.2 (35.4)	40.4 (38.3)	72.7 (38.3)	10.0 (27.4)	< 0.001

CU, Columbia University; MG, Massachusetts General Hospital; UM, University of Michigan Ann Arbor; TX, University of Texas Southwestern Medical Center; HDRS, Hamilton Depression Rating Scale.

eAppendix 10. Classifier Construction and Performance Estimation: Internal Validation with CAN-BIND

We reported the balanced accuracy achieved through k-fold cross-validation with CAN-BIND sample for various values of k (**eFigure 4**). Balanced accuracy ranged from 57.2% for 5-fold cross-validation to 64.0% for 40-fold cross-validation. With more folds, the model may provide a more comprehensive evaluation of its generalization capabilities as it is exposed to a greater variety of training scenarios. Consequently, the model may become more robust and perform better on unseen data.

© 2023 Schwartzmann B et al. *JAMA Network Open.*

eFigure 4. Balanced Accuracy in Internal Validation with CAN-BIND

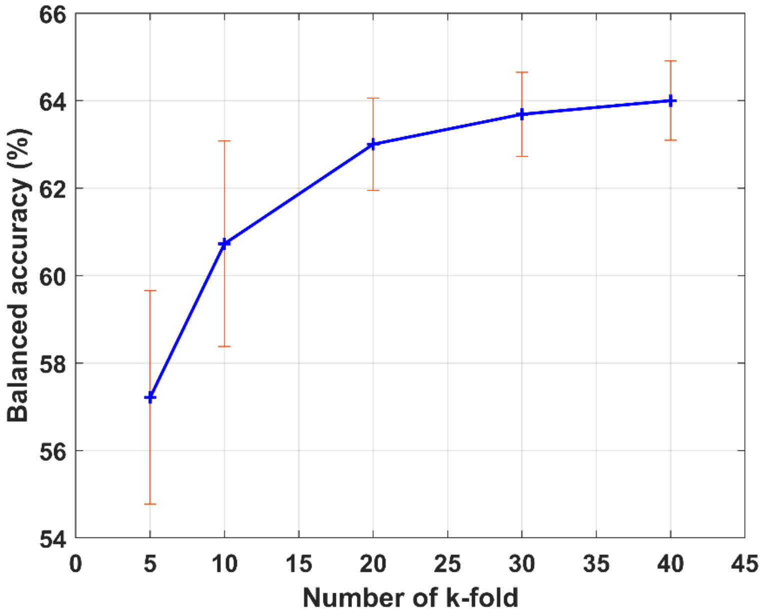

The figures display the balanced accuracy achieved through k-fold cross-validation with CAN-BIND sample for various values of k. The x-axis represents the k values, while the y-axis represents the balanced accuracy expressed in percentage. For each scenario, the balanced accuracy was calculated by averaging 10 iterations of the cross-validation procedure. The error bars depict the standard deviation across the 10 iterations.

eAppendix 11. Classifier Construction and Performance Estimation: External Validation with EMBARC

The model was tested on EMBARC placebo arm, but did not perform better than chance, yielding a balanced accuracy of 48.7% (95% CI [39.3, 58.0]; sensitivity, 50.0%, specificity, 47.3%) (**eTable 6**). To assess if there was a statistical difference in predicting the responses in the EMBARC sertraline and the placebo arms, we conducted a Chi-square test analysis. We set up a contingency table that represented the observed frequencies of correct and incorrect predictions in both EMBARC samples and computed the Chi-square test statistic (**eTable 7**). A statistical difference was observed between the predictions in EMBARC sertraline and placebo arms (p = 0.02).

© 2023 Schwartzmann B et al. *JAMA Network Open.*

eTable 6. External Validation With EMBARC Placebo Arm

	CU (n = 38)	MG (n = 21)	TX (n = 38)	UM (n = 21)	ALL (n = 118)
Sensitivity, % (95% CI)	42.9	37.5	55.6	83.3	50.0 (35.2-64.8)
Specificity, % (95% CI)	52.9	46.2	44.8	46.7	47.3 (35.9-58.7)
Balanced Accuracy, % (95% CI)	47.9	41.8	50.2	65.0	48.7 (39.3-58.0)

CI, Confidence Interval; CU, Columbia University; MG, Massachusetts General Hospital; TX, University of Texas Southwestern Medical Center; UM, University of Michigan Ann Arbor.

eTable 7. Contingency Table With Correct and Incorrect Predictions in EMBARC Sertraline and Placebo Arms

	EMBARC sertraline arm (n = 105)	EMBARC placebo arm (n = 118)
Correct predictions	67	57
Incorrect predictions	38	61

eAppendix 12. Impact of Symptoms Severity on Generalizability

To test whether the difference in symptoms severity between participants in CAN-BIND and EMBARC could have an impact on the generalizability of these results, we conducted an exploratory analysis by excluding participants in EMBARC who had low baseline HDRS scores. We decided to exclude participants in EMBARC who had a HDRS scores equal to or below 16 at baseline, which corresponds to a MADRS score below 20 (cutoff value for inclusion criteria in CAN-BIND). Out of the 223 participants in EMBARC, 75 in the sertraline arm and 88 in the placebo arm had a baseline HDRS score of 16 or higher. A model trained using CAN-BIND predicted the outcomes in this subgroup of the sertraline arm with a balanced accuracy of 64.1% (95% CI [53.2, 75.0]; sensitivity, 63.4%; specificity, 64.7%) (**eTable 8**). Once again, the model

© 2023 Schwartzmann B et al. *JAMA Network Open.*

did not predict the outcomes in the placebo arm, with attempt at prediction resulting in a balanced accuracy of 43.2% (95% CI [32.7, 54.2]; sensitivity, 38.3%; specificity, 48.1%) (**eTable 9**). Results for other cut-off values are reported in **eFigure 5.**

eTable 8. External Validation With EMBARC Sertraline Arm (Participants With HDRS >=16)

	CU (n = 21)	MG (n = 14)	TX (n = 27)	UM (n = 13)	ALL (n = 75)
Sensitivity, % (95% CI)	62.5	80.0	61.5	57.1	63.4 (48.7-78.2)
Specificity, % (95% CI)	40.0	88.9	64.3	50.0	64.7 (48.6-80.8)
Balanced Accuracy, % (95% CI)	51.2	84.4	62.9	53.6	64.1 (53.2-75.0)

CI, Confidence Interval; CU, Columbia University; MG, Massachusetts General Hospital; TX, University of Texas Southwestern Medical Center; UM, University of Michigan Ann Arbor.

eTable 9. External Validation With EMBARC Placebo Arm (Participants With HDRS >=16)

	CU (n = 29)	MG (n = 19)	TX (n = 26)	UM (n = 14)	ALL (n = 88)
Sensitivity, % (95% CI)	29.4	37.5	50.0	66.7	38.3 (21.9-54.8)
Specificity, % (95% CI)	50.0	54.6	45.0	45.4	48.1 (34.8-61.5)
Balanced Accuracy, % (95% CI)	39.7	46.0	47.5	56.1	43.2 (32.7-54.2)

CI, Confidence Interval; CU, Columbia University; MG, Massachusetts General Hospital; TX, University of Texas Southwestern Medical Center; UM, University of Michigan Ann Arbor.

eFigure 5. Impact of Symptoms Severity on Generalizability

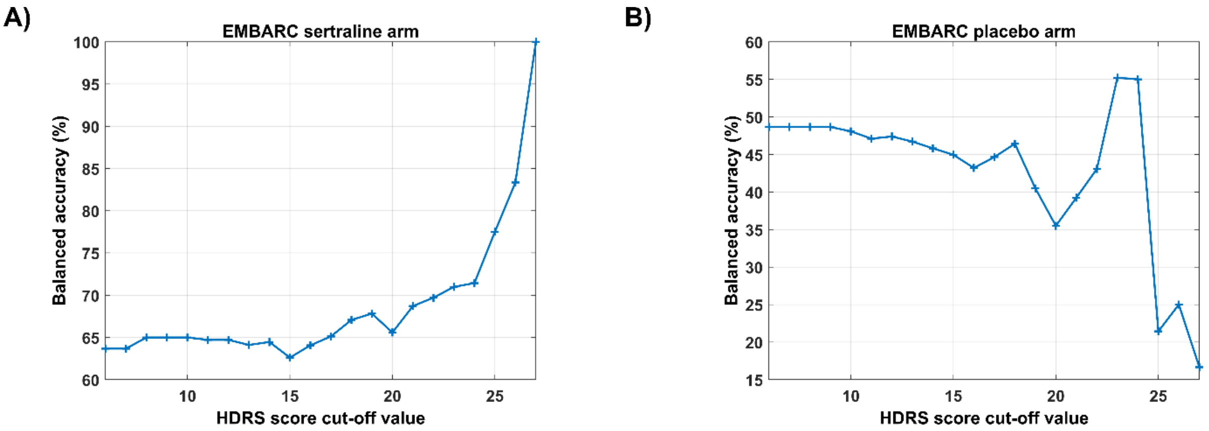

© 2023 Schwartzmann B et al. *JAMA Network Open.*

(A) The plot depicts the balanced accuracy obtained in EMBARC sertraline arm **(B)** The plot depicts the balanced accuracy obtained EMBARC placebo arm. **(A, B)** In both plots, x-axes represent the HDRS score cut-off values chosen to exclude EMBARC participants, y-axes the balanced accuracy obtained with the corresponding subgroup of participants.

eAppendix 13. Feature Importance

The process of randomly permuting the values of each of the 152 features in the external validation of the placebo arm of EMBARC resulted in a reduction in balanced accuracy ranging from -2.9% to 1.5% (**eFigure 6)**.

eFigure 6. Feature Importance in External Validation With EMBARC Placebo Arm

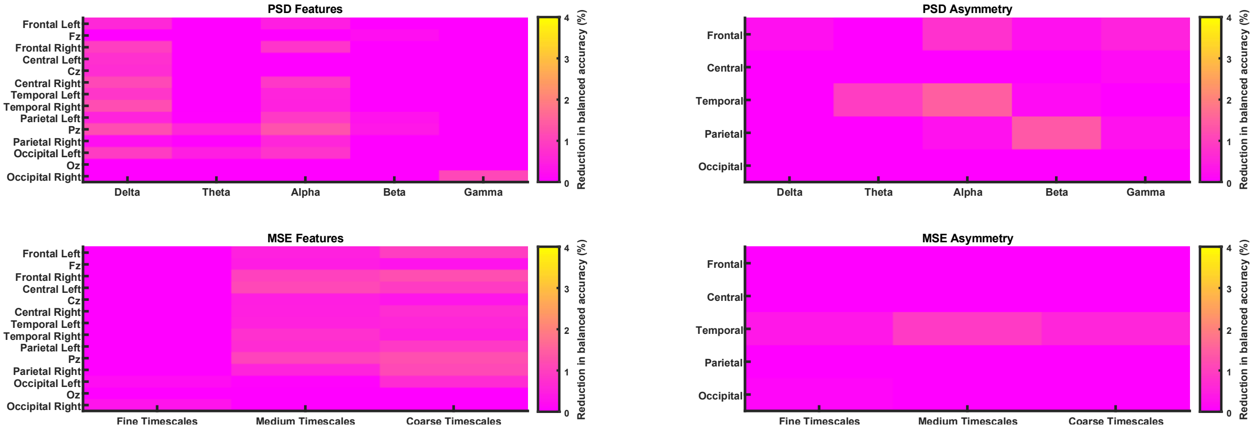

The figures display the feature importance results obtained in external validation with EMABRC placebo arm, where each matrix corresponds to the importance of power spectral density (PSD), PSD asymmetry, multiscale entropy (MSE), and MSE asymmetry features. The depicted features in pink colors indicate a low level of importance for the model, whereas the features represented in yellow colors indicate a stronger level of importance. Feature importance was evaluated based on the reduction in balanced accuracy in external validation with placebo arm of EMBARC. To enhance the clarity of the visualization, features exhibiting an increase in balanced accuracy were adjusted to 0%, indicating a lack of valuable information for predictive purposes (low level of importance). The display range was set from 0% to 4% to facilitate a more meaningful comparison with **Figure 1** in the manuscript.

© 2023 Schwartzmann B et al. *JAMA Network Open.*

eReferences

1. Kennedy, S. H. *et al.* Symptomatic and Functional Outcomes and Early Prediction of Response to Escitalopram Monotherapy and Sequential Adjunctive Aripiprazole Therapy in Patients With Major Depressive Disorder: A CAN-BIND-1 Report. *J. Clin. Psychiatry* **80**, 2236 (2019).

2. Lam, R. W. *et al.* Discovering biomarkers for antidepressant response: Protocol from the Canadian biomarker integration network in depression (CAN-BIND) and clinical characteristics of the first patient cohort. *BMC Psychiatry* **16**, 1–13 (2016).

3. Trivedi, M. H. *et al.* Establishing moderators and biosignatures of antidepressant response in clinical care (EMBARC): Rationale and design. *J. Psychiatr. Res.* **78**, 11–23 (2016).

4. Farzan, F. *et al.* Standardization of electroencephalography for multi-site, multi-platform and multi-investigator studies: Insights from the canadian biomarker integration network in depression. *Sci. Rep.* **7**, 1–11 (2017).

5. Kothe, C. A. E. & Jung, T.-P. Artifact removal techniques with signal reconstruction. at (2016).

6. Cheveigné, A. de. ZapLine: a simple and effective method to remove power line artifacts. *bioRxiv* 782029 (2019) doi:10.1101/782029.

7. Pion-Tonachini, L., Kreutz-Delgado, K. & Makeig, S. The ICLabel dataset of electroencephalographic (EEG) independent component (IC) features. *Data Br.* **25**, 104101 (2019).

8. Wong, T. T. Performance evaluation of classification algorithms by k-fold and leave-one-out cross validation. *Pattern Recognit.* **48**, 2839–2846 (2015).

9. Vabalas, A., Gowen, E., Poliakoff, E. & Casson, A. J. Machine learning algorithm validation with a limited sample size. *PLoS One* **14**, e0224365 (2019).

10. Varoquaux, G. Cross-validation failure : Small sample sizes lead to large error bars. *Neuroimage* **180**, 68–77 (2018).

© 2023 Schwartzmann B et al. *JAMA Network Open.*
