# Supplementary material for: Developing an Electroencephalography-Based Model for Predicting Response to Antidepressant Medication
Source: JAMA Netw Open. 2023 Sep 28;6(9):e2336094. doi: 10.1001/jamanetworkopen.2023.36094 (PMC10539986; doi:10.1001/jamanetworkopen.2023.36094)
Supplement: Supplement 2. — Data Sharing Statement [file jamanetwopen-e2336094-s002.pdf]

Data Sharing Statement

Schwartzmann. Developing an Electroencephalography-Based Model for Predicting Response to Antidepressant Medication. *JAMA Netw Open*. Published September 28, 2023. doi:10.1001/jamanetworkopen.2023.36094

Data
Data available: Yes
Data types: Deidentified participant data
How to access data: Data from CAN-BIND can already be accessed upon request here: https://www.braincode.ca/content/controlled-data-releases#dr008 Data from EMBARC can already be accessed upon request here: https://nda.nih.gov/edit_collection.html?id=2199
When available: beginning date: 06-07-2023

Supporting Documents
Document types: None

Additional Information
Who can access the data: Requests are managed by Brain-Code and NIHM
Types of analyses: Requests are managed by Brain-Code and NIHM
Mechanisms of data availability: Requests are managed by Brain-Code and NIHM
